# Predictors of Outcomes in Pediatric Acute Respiratory Distress Syndrome at a Tertiary Care Hospital

**DOI:** 10.7759/cureus.113998

**Published:** 2026-08-05

**Authors:** Hafeezullah Naikoo, Tahirah Khazar, Yawer N Shah, Naseer Yousuf Mir, Zubair Tramboo, Joziea Farooq, Tahira Ramzan

**Affiliations:** 1 Pediatrics, Sher-i-Kashmir Institute of Medical Sciences (SKIMS) Soura, Srinagar, IND; 2 Obstetrics and Gynecology, Sub District Hospital (SDH) Sogam, Srinagar, IND; 3 Pediatrics and Neonatology, Government Medical College (GMC) Handwara, Srinagar, IND; 4 Nursing, Government Medical College (GMC) Anantnag, Srinagar, IND; 5 Nursing, Sher-i-Kashmir Institute of Medical Sciences (SKIMS) Soura, Srinagar, IND

**Keywords:** ards, hypoxemic, lung injury, predictors, respiratory failure

## Abstract

Objectives: Acute respiratory distress syndrome (ARDS) in children remains a critical cause of morbidity and mortality worldwide, yet regional data from Kashmir are limited. This study aimed to evaluate the predictors of outcomes among pediatric ARDS cases admitted to a tertiary care center.

Methods: A prospective observational study was conducted over two years, enrolling 40 children diagnosed with ARDS at a pediatric department using a consecutive sampling method. Demographic characteristics, clinical features, laboratory trends, arterial blood gas (ABG) parameters, and severity scores (Lung Injury Score (LIS), Pediatric Sequential Organ Failure Assessment (pSOFA), and Pediatric Risk of Mortality III (PRISM III)) were analyzed to identify factors associated with disease progression and mortality.

Results: Most affected children were below six years of age, with male children comprising the majority. Fever, cough, tachypnea, crepitations, and hypoxemia were main clinical features. A significant rise in blood urea and creatinine emerged during progression, reflecting renal involvement. High total leukocyte count (TLC), neutrophilia, and inflammatory markers were associated with severe disease. Non-survivors exhibited markedly deranged ABG profiles-including lower pH, reduced PO₂, and elevated lactate-indicating uncompensated hypoxia and acidosis. Severity indices (LIS, pSOFA, and PRISM III) and oxygenation parameters significantly predicted mortality. Multiorgan dysfunction, particularly renal and hepatic impairment, was more frequent in non-survivors.

Conclusion: ARDS continues to carry high mortality in resource-limited settings. Younger age, elevated inflammatory and organ dysfunction markers, worsening ABG parameters, and higher severity scores were strongly associated with poor outcomes. Early identification of high-risk children, timely escalation of respiratory support, improved referral pathways, and strengthening of pediatric critical care services are essential to enhance survival in the region.

## Introduction

Acute respiratory distress syndrome (ARDS) continues to contribute significantly to the disease burden in pediatric critical care medicine. Pediatric ARDS (PARDS) is defined as a non-cardiogenic pulmonary edema characterized by an intense pulmonary inflammatory reaction, developing acutely within 12-48 hours in the context of a severe systemic illness resulting in hypoxemic respiratory failure [1]. Despite clinical advances, ARDS continues to be one of the most challenging disease processes encountered in intensive care practice, causing high mortality and disabling consequences among survivors [2]. In 2015, the first pediatric-specific ARDS definition was developed during the Pediatric Acute Lung Injury Consensus Conference (PALICC) to address the unique characteristics of PARDS and promote a better understanding of the syndrome [3]. Thousands of children die every year in pediatric intensive care units (PICUs) from PARDS. Using PALICC diagnostic criteria, PARDS has been recognized as a significant contributor to PICU admissions and mortality worldwide [4-6]. It carries high mortality in children admitted to PICUs worldwide. In a general PICU population in urban India, PARDS had a prevalence of 9.9% and mortality up to 51.4% [4].

## Materials and methods

This study was conducted in the Department of Pediatrics and Neonatology, Sher-i-Kashmir Institute of Medical Sciences (SKIMS) Soura, Srinagar, a tertiary care hospital in Jammu and Kashmir, from January 1, 2023, to January 1, 2025. This was a prospective cohort study. The population was selected from the PICU after taking proper informed written consent from parents/guardians of patients. The revised PALICC was used as a tool to diagnose patients with PARDS. Ethical approval was sought from the Ethical and Medical Review Board of the hospital on April 29, 2023, under reference number IEC SKIMS 086/2023 before enrollment of the cases. Informed consent in English, Urdu, and the native language was sought from the parents before enrollment in the study.

Study population

The study was conducted on patients in the age group of one month to 18 years.

Inclusion criteria

Inclusion criteria include all patients with PARDS between the ages of one month and 18 years.

Exclusion criteria

Exclusion criteria include all patients with underlying perinatal-related lung disease and all patients with cerebral palsy. The revised PALICC 2015 was used as a tool to diagnose patients with PARDS.

Operational definitions

Severe PARDS is defined by PALICC 2015 criteria as Oxygenation Index (OI) ≥ 16 or Oxygen Saturation Index (OSI) ≥ 12.3 for non-invasively/invasively ventilated patients. Acute kidney injury (AKI) is defined as per Kidney Disease: Improving Global Outcomes (KDIGO) criteria as doubling of baseline serum creatinine or oliguria < 0.5 mL/kg/h for >6 hours. Hepatic dysfunction is defined as alanine aminotransferase (ALT)/aspartate aminotransferase (AST) levels exceeding two times the upper limit of normal for age, or serum bilirubin ≥ 2.0 mg/dL. Multiple organ dysfunction syndrome (MODS) is defined as dysfunction in ≥2 organ systems according to the Pediatric Sequential Organ Failure Assessment (pSOFA) scoring framework [7]. Septic shock is defined as severe infection leading to cardiovascular dysfunction requiring vasoactive or inotropic support.

Data collection

All PICU admissions were screened for PARDS. The onset of PARDS was defined as the day on which the patient first met PALICC criteria, and severity grading was done after four hours of onset of PARDS. Demographic details, clinical history, physical examination, underlying illness, complete blood count (CBC), kidney function test (KFT), liver function test (LFT), coagulogram, septic markers, ventilation variables (FiO₂, SpO₂, PaO₂, and positive end-expiratory pressure (PEEP) for non-ventilated patients for SpO₂/FiO₂ (SF) and PaO₂/FiO₂ (PF) ratios and FiO₂, SpO₂, PaO₂, and mean arterial pressure (MAP) for ventilated patients for OSI and OI), laboratory and chest X-ray (CXR) data, and treatment details of all eligible cases were recorded in a predesigned proforma (Appendix). Severity indices at admission like the Pediatric Risk of Mortality (PRISM) III (includes cardiovascular and neurological vitals and biochemical and hematological tests), pSOFA (for the assessment of functional disability of various organ systems), and Lung Injury Score (LIS) (includes the PF ratio, degree of alveolar consolidation, and level of PEEP) were obtained using predefined scoring systems after four hours of onset of PARDS [7-9]. Complications related to treatment and ventilation were also noted.

Statistical analysis

Continuous variables were tested for normality using the Shapiro-Wilk test. Normally distributed continuous parameters were expressed as mean ± standard deviation and compared using Student’s t-test, whereas non-normally distributed variables were analyzed using the Mann-Whitney U test. Categorical variables were presented as counts (percentages) and compared using the Chi-squared test or Fisher's exact test as appropriate. All statistical analyses were conducted using SPSS Version 20.0 (IBM Corp., Armonk, NY, USA), with statistical significance set at p < 0.05.

## Results

The mean age of children with PARDS was 3.7 ± 3.2 years, with a marked predominance of children below six years of age (85%). Male children constituted 70% of cases. This distribution indicates that PARDS predominantly affects younger children in this setting, with a male preponderance. Cough (72.5%) and fever (55%) were the most common presenting symptoms, suggesting infection as the predominant precipitating factor. Features such as shock, skin manifestations, and abdominal pain were uncommon. Most children were critically ill at admission, with tachycardia, tachypnea, delayed capillary refill time, and significant hypoxemia. Respiratory examination uniformly revealed crepitations, reflecting diffuse alveolar involvement. Of the 40 children studied, 25 (62.5%) survived to discharge, while 15 (37.5%) died. Mortality was significantly higher among younger children and those with evidence of multiorgan dysfunction, severe hypoxemia, and higher severity scores.

Serial laboratory evaluation (Table 1) demonstrated that while hemoglobin and platelet counts were largely preserved initially, non-survivors had significantly higher leukocyte counts and lower platelet counts. Renal parameters showed a significant rise in blood urea and serum creatinine among non-survivors, indicating AKI. Liver enzymes (ALT and alkaline phosphatase (ALP)) were also significantly elevated in children who died, suggesting hepatic involvement. Inflammatory markers such as procalcitonin and lactate were significantly higher in non-survivors, reflecting severe systemic inflammation and tissue hypoxia.

**Table 1:** Comparison of Laboratory, Arterial Blood Gas, and Oxygenation Parameters Between Survivors and Non-survivors With PARDS Data are presented as mean ± standard deviation (mean ± SD) unless otherwise specified. Culture positivity is presented as frequency (n) and percentage (%). Continuous variables were compared using independent samples Student's t-test, while categorical variables were analyzed using Fisher's exact test. A p-value < 0.05 was considered statistically significant. PARDS: pediatric acute respiratory distress syndrome; HB: hemoglobin; TLC: total leukocyte count; PLT: platelet; Creat: creatinine; ALT: alanine aminotransferase; ALP: alkaline phosphatase; CRP: C-reactive protein; PCT: procalcitonin; PF: PaO₂/FiO₂; OI: Oxygenation Index

Variable	Non-survivors (n = 15) mean ± SD	Survivors (n = 25) mean ± SD	p-value
HB	10.95 ± 1.62	11.02 ± 1.29	0.316
TLC	15.42 ± 7.04	14.36 ± 4.04	0.01
PLT	182 ± 82.49	262.21 ± 111.43	0.02
Urea	45 ± 14.32	35.18 ± 9.60	0.01
Creat	1.1 ± 0.37	0.76 ± 0.33	<0.001
Bilirubin	0.53 ± 0.19	0.48 ± 0.20	0.440
ALT	105.42 ± 28.78	46.44 ± 15.15	<0.001
ALP	48.46 ± 42.93	39.66 ± 17.85	<0.001
Calcium	8.31 ± 1.03	8.76 ± 0.65	0.09
Culture positive/negative	4/11	1/24	-
CRP	99.22 ± 59.33	78.70 ± 60.55	0.302
PCT	18.45 ± 29.32	4.47 ± 6.68	0.02
pH	7.18 ± 0.09	7.31 ± 0.09	<0.001
PO_2_	55.33 ± 8.27	66.51 ± 12.65	0.004
PCO_2_	21.11 ± 2.77	49.8 ± 16.79	<0.001
HCO_3_	10.7 ± 4.9	18.93 ± 6.3	<0.001
Lactate	4.21 ± 2.64	1.92 ± 1.20	0.005
PF	88.67 ± 40.60	119.44 ± 45.02	0.03
OI	26.82 ± 15.12	11.89 ± 4.90	0.001

Comparison of arterial blood gas (ABG) values (Table 1) revealed that non-survivors had significantly lower pH, PaO₂, and bicarbonate levels, with markedly elevated lactate levels, indicating uncompensated metabolic acidosis and severe hypoxemia. Oxygenation parameters (Table 1) showed significantly lower PF ratios and higher OI values among non-survivors, highlighting the severity of lung injury and poor response to ventilatory support.

Severity scoring systems demonstrated strong prognostic relevance. As shown in Tables 2-4, mean LIS, pSOFA score, and PRISM III score were significantly higher in the non-survivor group. Higher scores were associated with increased mortality, prolonged mechanical ventilation, and longer ICU stay. These findings confirm the utility of these scoring systems in early risk stratification of children with PARDS.

**Table 2:** Comparison of Lung Injury Score (LIS) Between Survivors and Non-survivors Data are presented as frequency (n), percentage (%), and mean ± standard deviation (mean ± SD). Comparisons between groups were performed using independent samples Student's t-test. A p-value < 0.05 was considered statistically significant.

LIS score	Non-survivors, n (%)	Survivors, n (%)
7	1 (6.7%)	13 (52.0%)
8	1 (6.7%)	6 (24.0%)
9	2 (13.3%)	4 (16.0%)
10	2 (13.3%)	2 (8.0%)
11	2 (13.3%)	0 (0%)
12	0 (0%)	0 (0%)
13	2 (13.3%)	0 (0%)
14	3 (20.0%)	0 (0%)
15	1 (6.7%)	0 (0%)
17	1 (6.7%)	0 (0%)

**Table 3:** Comparison of Pediatric Sequential Organ Failure Assessment (pSOFA) Scores Between Survivors and Non-survivors Data are presented as frequency (n), percentage (%), and mean ± standard deviation (mean ± SD). Comparisons between groups were performed using independent samples Student's t-test. A p-value < 0.05 was considered statistically significant.

pSOFA score	Non-survivors, n (%)	Survivors, n (%)
4	0 (0%)	6 (24.0%)
5	1 (6.7%)	9 (36.0%)
6	0 (0%)	6 (24.0%)
7	2 (13.3%)	3 (12.0%)
8	2 (13.3%)	1 (4.0%)
9	4 (26.7%)	0 (0%)
11	2 (13.3%)	0 (0%)
14	2 (13.3%)	0 (0%)
15	1 (6.7%)	0 (0%)
25	1 (6.7%)	0 (0%)

**Table 4:** Comparison of Pediatric Risk of Mortality III (PRISM III) Scores Between Survivors and Non-survivors Data are presented as frequency (n), percentage (%), and mean ± standard deviation (mean ± SD). Comparisons between groups were performed using independent samples Student's t-test. A p-value < 0.05 was considered statistically significant.

PRISM III score	Non-survivors, n (%)	Survivors, n (%)
3	2 (13.3%)	12 (48.0%)
4	1 (6.7%)	4 (16.0%)
5	2 (13.3%)	2 (8.0%)
6	0 (0%)	3 (12.0%)
7	2 (13.3%)	2 (8.0%)
12	3 (26.7%)	2 (8.0%)
15	2 (13.3%)	0 (0%)
17	2 (13.3%)	0 (0%)
19	1 (6.7%)	0 (0%)

## Discussion

The present prospective study evaluated the clinical profile and predictors of outcome among children with PARDS admitted to a tertiary care center in Kashmir. PARDS remains a major cause of morbidity and mortality in PICUs, particularly in resource-limited settings. The study provides valuable regional data on demographic characteristics, clinical manifestations, laboratory abnormalities, and prognostic indicators associated with adverse outcomes.

The majority of children in our cohort were below six years of age, with a mean age of 3.7 years. Younger children constituted the most vulnerable group, which may be explained by immature immune responses, smaller physiological reserves, and increased susceptibility to severe respiratory infections. Similar age distributions have been reported in previous studies of PARDS, highlighting the disproportionate burden of disease among infants and young children [4-6]. Male predominance was observed in our study, a finding consistent with several earlier reports, although the underlying reasons remain uncertain and may involve both biological and sociocultural factors [5].

Fever and cough were the most common presenting symptoms, reflecting the predominance of infectious etiologies. Clinical examination revealed widespread respiratory involvement, with tachypnea, hypoxemia, and crepitations being common findings. These observations are consistent with the pathophysiology of PARDS, in which diffuse alveolar injury, pulmonary inflammation, and impaired gas exchange lead to progressive respiratory failure. The low oxygen saturation observed at admission in most patients underscores the importance of early recognition and timely respiratory support.

Serial laboratory evaluation demonstrated significant increases in blood urea and serum creatinine during the course of illness, suggesting the development of renal dysfunction in severe cases. AKI is increasingly recognized as an important complication of PARDS and is associated with poor outcomes. Elevated leukocyte counts and neutrophilia were also associated with disease progression, supporting the role of systemic inflammation in determining disease severity. Although ABG parameters did not show significant changes between baseline and worst recorded values in the overall cohort, marked differences were observed between survivors and non-survivors, indicating that deterioration in gas exchange and tissue perfusion is strongly associated with mortality.

The overall mortality rate in our study was 37.5%, which is comparable to reports from several developing countries but remains higher than rates reported from resource-rich settings. Differences in mortality across studies may be related to variations in disease severity, availability of pediatric intensive care facilities, ventilatory support strategies, and timing of referral. Delayed presentation to tertiary care centers may also contribute to poorer outcomes in our setting.

A significant finding of this study was the association between younger age and mortality. Children who died were substantially younger than survivors, suggesting that infants and toddlers possess limited physiological reserve to tolerate severe hypoxemia and systemic inflammation. In contrast, gender was not associated with outcome, indicating that disease severity rather than demographic characteristics determines survival following hospitalization.

Several laboratory markers demonstrated prognostic significance. Non-survivors had significantly higher total leukocyte counts, blood urea, serum creatinine, liver enzymes, procalcitonin levels, and serum lactate concentrations. These findings indicate that multiorgan dysfunction accompanies severe PARDS and contributes substantially to mortality. Elevated lactate levels may reflect impaired tissue perfusion and persistent hypoxia, while abnormalities in renal and hepatic function suggest systemic involvement beyond the lungs.

ABG analysis provided important prognostic information. Children who died had significantly lower pH and PaO₂ values together with markedly elevated lactate levels compared with survivors. These findings emphasize the importance of serial ABG monitoring in critically ill children, as persistent acidosis and hypoxemia may identify patients at increased risk of adverse outcomes. Careful monitoring of oxygenation and acid-base status remains essential for guiding ventilatory management and therapeutic interventions.

Severity scoring systems demonstrated strong predictive value in our cohort. Higher LIS, pSOFA score, and PRISM III score were all significantly associated with mortality. These scores evaluate different physiological domains but consistently identified children at highest risk of death. Similar findings have been reported in previous studies evaluating pSOFA and other severity indices in critically ill pediatric populations [7]. Routine use of severity scoring systems may therefore facilitate early risk stratification and guide clinical decision-making in children with PARDS.

Oxygenation parameters were also important predictors of outcome. Non-survivors had significantly lower PF ratios and higher OI values than survivors. These findings are consistent with previous studies demonstrating that worsening oxygenation impairment is strongly associated with mortality in PARDS [7]. Children requiring higher ventilatory support and exhibiting persistent oxygenation failure experienced poorer outcomes, emphasizing the need for aggressive monitoring and optimization of respiratory management.

This study identifies early laboratory indicators of poor prognosis, including rising blood urea, serum creatinine, liver enzymes, procalcitonin, and lactate, suggesting evolving multiorgan dysfunction in severe PARDS; confirms the strong prognostic utility of severity scoring systems (LIS, pSOFA, and PRISM III) in predicting mortality and ICU outcomes among children with PARDS in resource-limited settings; and highlights the prognostic significance of oxygenation parameters (PF ratio and OI).

Limitations

As the study was conducted at a single tertiary care center, this may limit the generalizability of the findings to other healthcare settings. Second, the relatively small sample size may have reduced the statistical power to identify less common predictors of outcome. Third, the observational design precludes establishing causal relationships between identified risk factors and clinical outcomes. Additionally, variations in treatment practices and supportive care during hospitalization could have influenced patient outcomes. Furthermore, due to sample size constraints, univariable associations between laboratory/physiological markers and mortality could not be fully adjusted for all clinical confounders (such as baseline nutritional status, co-morbid immunodeficiency, or specific adjunctive therapies like steroids). Larger multicenter cohorts are required to perform robust multivariable adjustments.

## Conclusions

The study highlights that PARDS continues to be a life-threatening clinical condition with substantial mortality, predominantly affecting younger pediatric patients. Early clinical severity, deranged laboratory parameters, and high physiological scoring indices (LIS, pSOFA, and PRISM III) were strongly associated with poor outcomes. The findings reinforce that timely recognition of high-risk children and prompt escalation of respiratory and critical care support may improve survival. Continuous severity scoring, frequent monitoring of physiological markers, and early intervention in deteriorating patients appear essential in improving the prognosis of PARDS in resource-limited critical care settings.

## Appendices

**Table 5:** Case Record Proforma PARDS diagnosed according to the Revised Pediatric Acute Lung Injury Consensus Conference (PALICC) criteria. Severity scores (LIS, pSOFA, and PRISM III) should be calculated within 4 hours of PARDS diagnosis. Laboratory investigations and arterial blood gas measurements should be repeated according to the treating physician's clinical judgment. TLC: total leukocyte count; ALT: alanine aminotransferase; AST: aspartate aminotransferase; ALP: alkaline phosphatase; CRP: C-reactive protein; HFNC: high-flow nasal cannula; CPAP: continuous positive airway pressure; NIV: non-invasive ventilation; PEEP: positive end-expiratory pressure; MAP: mean arterial pressure; PF: PaO₂/FiO₂; SF: SpO₂/FiO₂; LIS: Lung Injury Score; pSOFA: Pediatric Sequential Organ Failure Assessment; PRISM: Pediatric Risk of Mortality; PICU: pediatric intensive care unit; PARDS: pediatric acute respiratory distress syndrome

Variable	Details
Study ID	___
Hospital registration no.	___
Date of admission	__/__/__
Date of enrollment	__/__/__
Age	___ months/years
Sex	☐ Male ☐ Female
Weight (kg)	___
Residence	___
Referred from	___

Complaint	Present
Fever	☐ Yes ☐ No
Cough	☐ Yes ☐ No
Breathlessness	☐ Yes ☐ No
Fast breathing	☐ Yes ☐ No
Poor feeding	☐ Yes ☐ No
Vomiting	☐ Yes ☐ No
Convulsions	☐ Yes ☐ No
Altered sensorium	☐ Yes ☐ No
Abdominal pain	☐ Yes ☐ No
Rash	☐ Yes ☐ No
Shock	☐ Yes ☐ No
Other	___

Variable	Details
Duration of illness	___ days
Previous hospitalization	☐ Yes ☐ No
Previous mechanical ventilation	☐ Yes ☐ No

Condition	Present
Congenital heart disease	☐ Yes ☐ No
Chronic lung disease	☐ Yes ☐ No
Immunodeficiency	☐ Yes ☐ No
Malignancy	☐ Yes ☐ No
Other	___

Parameter	Value
Temperature	___ °C
Heart rate	___ /min
Respiratory rate	___ /min
Blood pressure	___ mmHg
Capillary refill time	___ sec
SpO₂	___ %
Glasgow Coma Scale	___

Finding	Present
Crepitations	☐ Yes ☐ No
Wheeze	☐ Yes ☐ No
Retractions	☐ Yes ☐ No
Cyanosis	☐ Yes ☐ No
Decreased air entry	☐ Yes ☐ No
Other findings	___

Etiology	Present
Pneumonia	☐ Yes ☐ No
Sepsis	☐ Yes ☐ No
Aspiration	☐ Yes ☐ No
Viral infection	☐ Yes ☐ No
Trauma	☐ Yes ☐ No
Near drowning	☐ Yes ☐ No
Other	___

Final diagnosis
___
___

Parameter	Value
Hemoglobin	___
TLC	___
Platelet count	___

Parameter	Value
Blood urea	___
Serum creatinine	___

Parameter	Value
Total bilirubin	___
ALT	___
AST	___
ALP	___

Parameter	Value
Sodium	___
Potassium	___
Calcium	___

Parameter	Value
CRP	___
Procalcitonin	___
Blood culture	☐ Positive ☐ Negative
Organism isolated	___

Parameter	Admission	Worst value
pH		
PaO₂		
PaCO₂		
HCO₃		
Lactate		

Variable	Findings
Bilateral infiltrates	☐ Yes ☐ No
Quadrants involved	☐ One ☐ Two ☐ Three ☐ Four
Pleural effusion	☐ Yes ☐ No
Comments	___

Mode	Used
Nasal cannula	☐ Yes ☐ No
HFNC	☐ Yes ☐ No
CPAP	☐ Yes ☐ No
NIV	☐ Yes ☐ No
Mechanical ventilation	☐ Yes ☐ No

Parameter	Value
FiO₂	___
PEEP	___
MAP	___
SpO₂	___
PaO₂	___
PF ratio	___
SF ratio	___
Oxygenation Index (OI)	___
Oxygen Saturation Index (OSI)	___

Component	Score
Chest X-ray	
Hypoxemia	
PEEP	
Compliance	
Total LIS	

Organ system	Score
Respiratory	
Cardiovascular	
Neurological	
Liver	
Kidney	
Platelets	
Total pSOFA	

Variable	Score
Total PRISM III score	___

Treatment	Details
Antibiotics	___
Antivirals	___
Steroids	☐ Yes ☐ No
Vasopressors	☐ Yes ☐ No
Mechanical ventilation	☐ Yes ☐ No
Duration of ventilation	___ days
Prone ventilation	☐ Yes ☐ No
Renal replacement therapy	☐ Yes ☐ No
Other interventions	___

Complication	Present
Septic shock	☐ Yes ☐ No
Acute kidney injury	☐ Yes ☐ No
Liver dysfunction	☐ Yes ☐ No
Pneumothorax	☐ Yes ☐ No
Ventilator-associated pneumonia	☐ Yes ☐ No
Disseminated intravascular coagulation	☐ Yes ☐ No
Multiorgan dysfunction syndrome	☐ Yes ☐ No
Other	___

Variable	Details
Duration of PICU stay	___ days
Duration of hospital stay	___ days
Outcome	☐ Survived ☐ Died
Cause of death (if applicable)	___
Date of discharge/death	__/__/__
